# The Possible Role of Neutrophils in the Induction of Osteoclastogenesis

**DOI:** 10.1155/2019/8672604

**Published:** 2019-09-15

**Authors:** Carolyn G. J. Moonen, Teun J. de Vries, Patrick Rijkschroeff, Patrice E. Poubelle, Elena A. Nicu, Bruno G. Loos

**Affiliations:** ^1^Department of Periodontology, Academic Centre for Dentistry Amsterdam (ACTA), University of Amsterdam and Vrije Universiteit Amsterdam, Amsterdam, Netherlands; ^2^Department of Medicine, Centre de Recherche en Rhumatologie et Immunologie (CRRI), Centre Hospitalier de l'Université Laval (CRCHUL), Faculty of Medicine, Université Laval, Québec, QC, Canada; ^3^CMI Dr. Opris M.I., Sibiu, Romania

## Abstract

The ligand of the receptor activator of NF-*κ*B (RANKL) is a key molecule in the formation of osteoclasts, the key cells that cause the disease-associated alveolar bone resorption in periodontitis. We hypothesized that polymorphonuclear leukocytes (PMNs), found as the most prominent cells of inflamed periodontal tissues, could play an important role in providing signals to trigger osteoclastogenesis and thus activating pathological bone resorption in periodontitis. RANKL expression was investigated on circulatory PMNs (cPMNs) and oral PMNs (oPMNs) taken from both controls and periodontitis patients. On average, 2.3% and 2.4% RANKL expression was detected on the cPMNs and oPMNs from periodontitis patients, which did not differ significantly from healthy controls. Since cPMNs may acquire a more osteoclastogenesis-facilitating phenotype while migrating into the inflamed periodontium, we next investigated whether stimulated (with LPS, TNF-*α*, or IL-6) cPMNs have the capacity to contribute to osteoclastogenesis. Enduring surface expression of RANKL for short-lived cells as cPMNs was achieved by fixating stimulated cPMNs. RANKL expression on stimulated cPMNs, as assessed by flow cytometry and immunohistochemistry, was limited (6.48 ± 0.72%, mean expression ± SEM) after 24 and 48 hours of stimulation with LPS. Likewise, stimulation with TNF-*α* and IL-6 resulted in limited RANKL expression levels. These limited levels of expression did not induce osteoclastogenesis when cocultured with preosteoclasts for 10 days. We report that, under the aforementioned experimental conditions, neither cPMNs nor oPMNs directly induced osteoclastogenesis. Further elucidation of the key cellular players and immune mediators that stimulate alveolar bone resorption in periodontitis will help to unravel its pathogenesis.

## 1. Introduction

Periodontitis is a chronic inflammatory disease of the tooth-supporting tissues (e.g., the periodontium). The chronic inflammatory cell infiltration of the periodontal soft tissues is accompanied by osteoclast-induced alveolar bone resorption, the hallmark of periodontitis progression [1, 2]. Osteoclasts are derived from monocyte/macrophage precursors and regulate bone resorption. Monocyte differentiation into osteoclasts requires the activation of their RANK receptors that recognize activator NF-kappa B-ligand (RANKL) [3]. Additionally, macrophage colony-stimulating factor (M-CSF) is needed to trigger differentiation in osteoclast cultures [4]. To differentiate into (pre-)osteoclasts, monocytes likely receive their RANKL differentiation signal from cell-cell interactions [5]. Expression of RANKL has been reported on a wide variety of cells of the periodontium, including T cells, B cells [6], and periodontal ligament and gingival fibroblasts [7]. Alveolar bone osteocytes also express RANKL, and it has recently been demonstrated that especially osteocyte-expressed RANKL could be crucial in the initiation of periodontitis as demonstrated in a RANKL knock-out mouse model with a targeted disruption of RANKL in osteocytes [8], reviewed by De Vries and Huesa [9]. RANKL in humans is expressed in three different forms: the primary secreted soluble form sRANKL, the cell membrane-bound and transmembrane RANKL (mRANKL), and a truncated ectodomain moiety cleaved from the cell-bound form [10].

The host inflammatory response in periodontitis is induced by the constant interaction occurring between host cells and the biofilm present at the roots of the teeth. An aberrant host response creates a shift in the ecosystem where Gram-negative bacteria can thrive, resulting in a dysbiotic microflora, reviewed by Lamont et al. [11]. LPS is a cell wall component of Gram-negative bacteria and is widely considered to be a potent stimulator of innate host defenses. One of the major pathogens associated with periodontitis is *Porphyromonas gingivalis.* Even at low colonization levels, provided that the ecosystem is favorable, *P. gingivalis* can disrupt the homeostasis of the commensal dental biofilm and can enhance a dysbiotic microflora [11]. This shift in the microfloral environment can aggravate inflammatory immune responses, including the production of proinflammatory cytokines, in a range of host cells such as gingival fibroblasts, gingival epithelial cells, monocytes, macrophages, and polymorphonuclear leukocytes (PMNs) [12–15]. Several proinflammatory cytokines that are elevated in periodontal disease, such as tumor necrosis factor alpha (TNF-*α*) and interleukin- (IL-) 6, have been shown to stimulate osteoclastogenesis [16–19]. These proinflammatory cytokines can, in turn, promote monocyte differentiation into preosteoclasts and eventually trigger the activation of osteoclasts independently of the RANKL pathway [19]. Elevated RANKL release or expression is possibly caused by bacterial products such as LPS, which on its own can also enhance RANKL expression [20]. LPS as an initial stimulator can evoke inflammatory responses and can subsequently stimulate or enhance osteoclast formation, leading to both elevated numbers of osteoclasts and increased osteoclastic activity. In periodontitis, this potentially leads to irreversible alveolar bone resorption and, eventually, tooth loss.

Our group described that bacterial priming of the osteoclastogenesis-inducing cells residing in the periodontium, such as the periodontal ligament fibroblasts, alters the potential for osteoclast formation *in vitro* [14, 21]. Furthermore, we also found that gingival fibroblasts play a crucial role in osteoclastogenesis when cultured with monocytes. Next to their role in osteoclastogenesis, they also facilitate the survival, retention, and selective proliferation of lymphocytes [22]. Dutzan et al. confirmed the distinct cellular composition of periodontitis lesions when compared to uninflamed healthy gingiva [23]. As such, periodontal lesions show a substantial infiltration of innate immune responders, i.e., PMNs.

PMNs originate in the bone marrow and are found in circulating blood (further referred to as circulatory PMNs (cPMNs)) in numbers between 2.5 and 7.5 × 10^9^/L. These cell numbers can increase in a chronic inflammatory state such as in periodontitis, morbid obesity, diabetes mellitus, and atherosclerotic vascular disease [24–29]. Although resting cPMNs have a short lifespan (6-8 hours in circulation), stimulated cPMNs have been shown to have an extended lifespan (several days) and are capable of synthesizing considerable amounts of proteinaceous and lipid immune mediators, which are important in inflammatory processes [30, 31]. Although high numbers of PMNs have been found at sites of bone erosion [32], their impact on the differentiation of monocytes into preosteoclasts and mature osteoclasts remains unclear.

PMNs are also found both in the oral cavity and saliva (further referred to as oral PMNs (oPMNs)). The gingival crevice (sulcus) is identified as the main point of entrance for oPMNs transiting towards the oral cavity. However, their transmigration through all other mucosal tissues has also been found [33]. Under healthy conditions, approximately 30,000 oPMNs per minute have been shown to enter the oral cavity through the crevices around the teeth; however, the number of oPMNs entering the oral cavity increases by a factor of 4 in cases of gingival inflammation (i.e., gingivitis) or periodontitis [34]. In contrast to cPMNs which exist in the almost-sterile circulatory system, the extracellular environment of oPMNs consists of salivary factors, oral bacteria, shed epithelial cells, and cell debris. Accordingly, oPMNs were shown to have exhausted capacity for efficient chemotaxis which may be the result of migration through the oral tissues into the oral cavity and they produce more ROS and NETs than cPMNs [34–36]. To date, RANKL expression on oPMNs has not yet been investigated. Interestingly, the mRANKL expression has been reported on cPMNs and synovial fluid-derived PMNs from rheumatoid arthritis patients [37–39]. Moreover, the expression of mRANKL in these cPMNs appears upregulated in the presence of bacterial lipopolysaccharide (LPS). By fixing cPMNs and adding them to live osteoclast precursors, the transmembranic RANKL was shown to induce differentiation of these preosteoclasts [37].

PMNs are one of the most prominent cells in periodontitis lesions where they are often activated or in a hyperactive state [40, 41]. Therefore, PMNs could conceivably play an important role in providing signals to trigger osteoclastogenesis activating pathological bone resorption in periodontitis. Through this study, we attempted to validate the aforementioned hypothesis in two ways. In part A, we investigated whether oPMNs, as a model representing the activated PMNs from periodontitis lesions, express RANKL and whether they can be primed and activated in response to the continuous presence of extracellular stimulants (saliva, oral bacteria, shed epithelial cells, and cell debris) that are present in the gingival sulcus and oral cavity. To accomplish this, RANKL expression was investigated in the cPMNs and oPMNs of both healthy controls and periodontitis patients. In part B of this study, we investigated whether cPMNs, after activation by the immunological modulators LPS, IL-6, or TNF-*α*, have the capacity to contribute to osteoclast formation via RANKL expression as previously published by Chakravarti et al. [37].

## 2. Materials and Methods

### 2.1. Study Design

This study consisted of two parts. Part A of this study, investigating RANKL expression on cPMNs and oPMNs, was carried out at the Department of Periodontology at the Academic Centre for Dentistry Amsterdam (ACTA), Amsterdam, The Netherlands. In part B of this study, the *in vitro* capacity of cPMNs to induce osteoclastogenesis was investigated at the Department of Medicine, Université of Laval (Québec, Canada) and experiments were performed as previously described [37].

### 2.2. Part A

#### 2.2.1. Study Population

Control subjects (*n* = 13) without periodontitis were recruited among individuals scheduled for regular dental check-ups at the educational practice at ACTA. Controls had to be at least 25 years of age. To ensure controls were not having periodontitis, the following criteria needed to be fulfilled: (i) a maximum CPITN (Community Periodontal Index for Treatment Needs) score [42] of 3 in any of the 6 possible sextants (corresponding to a maximum pocket depth of 4-5 mm), (ii) no gingival recession at the sites having a pocket depth of 4 or 5 mm, and (iii) no alveolar bone loss visible on recent (<1 year ago) bite-wing radiographs.

Periodontitis patients (*n* = 9) in this part of the study were recruited among those who were referred to the Department of Periodontology at ACTA for diagnosis and treatment. Full mouth periodontal charts (except attachment level measurements) were made by various periodontists of the Department of Periodontology (ACTA) and were retrieved from the dental records. Periodontitis was defined based on the criteria for periodontitis as previously agreed upon: the presence of proximal attachment loss of at least 3 mm in at least 2 nonadjacent teeth [43]. Alveolar bone loss was confirmed on recent X-rays (vertical bite-wings or periapical radiographs less than 1 year old). Periodontitis patients had to be at least 36 years of age and had not received periodontal treatment in the year preceding the study.

The following exclusion criteria were applied to the whole study population: American Society of Anesthesiologists (ASA) classification of ≥2 [44], pregnancy and lactation currently or in the past year, systemic disease, autoimmune disease or immunodeficiency, use of antibiotics or immune-influencing medication in the past year, acute bacterial or viral infections, oral wounds, and current or past chemotherapy.

The study was approved by the Medical Ethical Committee of the Amsterdam University Medical Centre, The Netherlands (2012-210#B2012406). Written informed consent and a questionnaire were obtained from all participants, and all experiments were conducted according to Dutch law.

#### 2.2.2. cPMN Collection and Isolation for Part A of This Study

Isolation of cPMNs was performed as previously described [36]. Venous blood (2 × 10 mL) from controls (*n* = 13) and periodontitis patients (*n* = 9) was obtained in lithium heparin tubes (Vacuette® Heparin tubes, Greiner Bio-One, Alphen a/d Rijn, The Netherlands). Blood was diluted 1 : 1 in 1% PBS citrate (pH 7.4). Subsequently, 25 mL of the diluted blood was carefully layered on top of 15 mL Lymphoprep (Axis-shield PoC AS, Oslo, Norway). After centrifugation (800 RCF, 30 min, RT, no brake), the supernatant above the red cell layer was discarded, after which remaining erythrocytes were lysed in cold lysis buffer (NH_4_Cl (1.5 M), NaHCO_3_ (100 mM), disodium EDTA (1 mM), all Sigma-Aldrich, Merck, Darmstadt, Germany, 10x diluted in sterile Milli-Q (MQ) water). Immediately after erythrocyte lysis, the cPMN pellet was washed twice in cold PBS (Gibco, Thermo Fisher Scientific, Paisley, Scotland, UK) and recovered in a culture medium (phenol red-free, Roswell Park Memorial Institute (RPMI) 1640, Gibco). All samples were handled on the same day without delay.

#### 2.2.3. oPMN Collection and Isolation

oPMNs were isolated as previously described [45, 46]. Controls (*n* = 13) and periodontitis patients (*n* = 9) rinsed the oral cavity 4 times with 10 mL 0.9% NaCl solution (Versylene®, Fresenius Kabi, Sèvres, France) for 30 seconds with 4-minute intermission periods. Per subject, the collected samples were pooled and centrifuged (500 RCF, 10 min, 4°C), and finally, the pellet was recovered in 40 mL PBS. The filtration protocol consisted of 4 filtrations with 70.0, 40.0 (Greiner Bio-One), 31.5, and 10.5 micrometers (*μ*m) nylon meshes (Vlint, Nedfilter, Almere, The Netherlands) to exclude epithelial cells and cell debris. The filtrated fraction was centrifuged (500 RCF, 10 min, 4°C), washed in cold PBS, and suspended in a phenol red-free culture medium. All samples were handled on the same day without delay.

#### 2.2.4. Flow Cytometry Analysis

The expression of RANKL on cPMNs and oPMNs from controls and periodontitis patients was analyzed using flow cytometry. Directly after isolation, PMNs were stained with either the mouse anti-human surface RANKL (PE conjugated, clone 12A380, R&D Systems, Minneapolis, MN, USA) or the isotype control IgG_1_ (PE conjugated, BD Biosciences, Franklin Lakes, NJ, USA). All cells were stained with anti-human CD16 (APC conjugated, clone 3G8, BD Biosciences) and anti-human CD66b (FITC conjugated, clone G10F5, BD Biosciences) as a PMN marker to assess PMN purity [46]. Flow cytometric analysis was performed on the Accuri C6 flow cytometer (BD Biosciences), where at least 1,000 cells were analyzed. The gating strategy employed is shown in Supplementary [Supplementary-material supplementary-material-1]. The PMNs were gated according to their relative size (Forward Scatter, FSC) and granularity (Side Scatter, SSC) and characteristic CD16 and CD66b expression [46]. In the live gating (encircled in red, Supplementary [Supplementary-material supplementary-material-1]), RANKL expression was quantified. This expression was corrected for the nonspecific binding of isotype control antibodies (IgG_1_-PE).

### 2.3. Part B

#### 2.3.1. cPMN Collection and Isolation

Volunteers were recruited among subjects attending the blood donation facility at the Centre de Recherche du Centre Hospitalier de l'Université Laval (Québec, Canada). The study was approved by the institutional review board of the Université Laval, Québec, Canada. Volunteers signed a written informed consent in accordance with the Declaration of Helsinki.

cPMNs were isolated from systemically healthy blood donors (*n* = 24 in total), all nonsmokers with an average age of 43 ± 10 years. Isolation of cPMNs was performed as previously described [37]. Venous blood (500 mL) was collected in 10 mL citrate-coated tubes (Thermo Fisher Scientific, Eugene, Oregon, USA). Per subject, blood was distributed in 50 mL tubes and after centrifugation (300 RCF, 10 min, at room temperature (RT), acceleration 7, deceleration 7), platelet-rich plasma was removed. After 30 minutes of red blood cell sedimentation with dextran (10 mL 2% dextran from *Leuconostoc* spp., 1 M HEPES, 0.16 M CaCl_2_, 10% Hanks' balanced salt solution (HBSS, 10x, without phenol red, sodium bicarbonate or calcium and magnesium, Multicell by Wisent Inc., St. Bruno, Québec, Canada) in sterile MQ, pH 7.4), the supernatant was transferred to a new tube and layered on Ficoll-Paque (Multicell, Wisent Inc.). After density gradient centrifugation (800 RCF, 30 min, no brake, at room temperature), the supernatant containing serum and Ficoll was removed, PBMCs were transferred to another tube for further isolation (see below), and contaminating erythrocytes were lysed by hypotonic lysis using sterile MQ water. After maximally 20 seconds of lysis in MQ, cells were recovered in 40 mL 10x HBSS. Finally, cPMNs were washed and suspended in a culture medium (RPMI 1640, +10% fetal bovine serum, 1% penicillin-streptomycin, all Wisent Inc.). Cell counts were routinely assessed by trypan blue staining using a hemocytometer.

#### 2.3.2. Isolation of Monocytes from PBMCs

In parallel to cPMN isolation, PBMCs were isolated from the same blood donor, on the same day and without delay. PBMCs were collected after Ficoll-Paque density gradient centrifugation. The percentage of monocytes present (approximately 10-30%) was determined by flow cytometry (BD FACSVerse™) after anti-human CD14 staining (clone M5E2, eBioscience Inc. by Thermo Fisher). PBMCs were seeded in plastic culture flasks at a density of 1 × 10^6^ monocytes/mL (polystyrene, nonpyrogenic cell culture flasks, Falcon®, Corning, New York, NY, USA). This allowed the attachment of monocytes (37°C, 5% CO_2_). Since peripheral blood lymphocytes (PBLs) are nonadhering cells, PBLs were removed by aspiration of the medium after 2 hours. Finally, monocytes were collected after detachment with Accutase (Wisent Inc.) and counted by trypan blue using a hemocytometer.

#### 2.3.3. cPMN Stimulation

To measure RANKL expression, the cPMNs were stimulated with LPS, TNF-*α*, or IL-6. cPMNs (5 × 10^6^ cells/mL) were incubated in 12-well plates with a range of concentrations of LPS (100, 500, and 1,000 ng/mL, *Escherichia coli*, 0111: B4; Sigma-Aldrich, Oakville, ON, Canada), IL-6 (10, 100, 500, or 1,000 ng/mL, R&D Systems), or TNF-*α* (recombinant human, *E. coli*-derived, 10, 50, or 100 ng/mL, R&D Systems, Oakville, ON, Canada). Prior to the addition, LPS was sonicated for 5 minutes and kept at room temperature until use. Granulocyte-macrophage colony-stimulating factor (GM-CSF, 1 nM, Peprotech, Rocky Hill, NJ, USA) was added to all conditions in order to increase cPMN viability.

#### 2.3.4. cPMN Enrichment

After 24 and 48 hours of stimulation, viable cPMNs were enriched by discontinuous Percoll (1.1309 gram/mL, GE Healthcare, Biosciences, Mississauga, ON, Canada) density gradient centrifugation as previously described [47]. Briefly, equal gradients of 31%, 42%, and 51% Percoll in a 10x RPMI medium (RPMI-1640 containing L-glutamine and phenol red, supplemented with 1.19 mM sodium bicarbonate, 1.2 mM HEPES, and 1% BSA, all Wisent Inc.) were layered and cell suspensions were carefully layered on top of these gradients. All gradient solutions were kept at 4°C. After centrifugation (610 RCF, 28 minutes, 4°C), the pellet containing viable cPMNs was collected. Finally, cPMNs were washed in PBS, recovered in a culture medium, and counted with a hemocytometer.

#### 2.3.5. Fixation of cPMNs

After stimulation, cPMNs were fixated for 15 minutes at room temperature with 2% paraformaldehyde (0.67 M PFA in PBS, Wisent Inc.) and washed three times in PBS [37].

#### 2.3.6. Immunohistochemical RANKL Staining

cPMNs, either stimulated with LPS (100 ng/mL in PBS, 30 minutes 37°C) or unstimulated, were centrifuged onto glass slides using a Cytospin-2 centrifuge (Shandon Southern Products, Astmoor, U.K.). Image-iT® FX Signal Enhancer (Invitrogen, Thermo Fisher Scientific Inc.) was used to block nonspecific binding sites. After washing with PBS, cells were incubated with the primary anti-human antibody RANKL (Human TRANCE/TNFSF11/RANKL Monoclonal Mouse IgG_2B_ Clone 70525, R&D Systems) or mouse IgG_2B_ control (clone MPC11, R&D Systems) for 1 hour at room temperature. This was followed by a secondary antibody staining (Alexa Fluor 488 goat anti-human IgG, Invitrogen). After 1 hour of incubation in the dark, slides were washed twice with PBS. Nuclei of the cells were stained with propidium iodide (PI) containing a fluorescent vectashield (Vectashield with PI, H-1300, Vector Laboratories Inc. CA, Burlingame, CA, USA). The analysis was performed using confocal microscopy with a 20x objective and a 4x zoom (Leica Microsystems TCS, SP2, Wetzlar, Germany) for multidimensional imaging of the cells. Leica confocal analysis software program (version 2.6, Leica Microsystems) was used for imaging.

#### 2.3.7. Flow Cytometric Analysis

RANKL expression of cPMNs was analyzed using flow cytometry as described before [37], comparing the expression of unstimulated and stimulated cPMNs. After blocking (Human Trustain FcX™, BioLegend, San Diego, CA, USA), cPMNs were stained with either the mouse anti-human surface RANKL (PE conjugated, clone 12A380, Santa Cruz Biotechnology, Mississauga, ON, Canada) or the isotype control IgG_1_ (PE conjugated, BD Biosciences, Mississauga, ON, Canada). All cells were stained with anti-human CD66b (FITC conjugated, clone G10F5, eBioscience Inc. by Thermo Fisher) as a cPMN marker to assess PMN purity [46]. Flow cytometric analysis was performed using a flow cytometer FACSVerse™ (BD Biosciences) where at least 5,000 cells were analyzed. The gating strategy employed for flow cytometric analysis is shown in Supplemental [Supplementary-material supplementary-material-1]. The cells were gated according to their relative size (Forward Scatter, FSC) and granularity (Side Scatter, SSC). RANKL expression was analyzed for live, CD66b-positive cells and was corrected for isotype IgG_2B_ expression. Flow cytometry data were analyzed using the FACSuite software (version 6.1.3, BD Biosciences).

#### 2.3.8. Osteoclastogenesis

Osteoclastogenesis assays with activated, fixed cPMNs in coculture with preosteoclasts was performed as previously described [37]. Briefly, monocytes (250,000 per well) were seeded in duplicate and allowed to attach for 2 days in 48-well plates in triplicate. Monocytes were cultured in an osteoclastogenic RPMI medium containing 25 ng/mL M-CSF (Human M-CSF recombinant protein, eBioscience by Invitrogen) and 80 ng/mL RANKL (Human sRANKL ligand, Peprotech). After 2 days, the cytokine- or LPS-activated and fixed cPMNs (5 × 10^5^ per well) were cocultured with monocytes in the presence of 25 ng/mL M-CSF. Control conditions contained 80 ng/mL RANKL. Cultures were refreshed every 3-4 days and maintained for 10 days (37°C, 5% CO_2_).

#### 2.3.9. Osteoclast Staining Using the Fluorescence ELF-97 TRACP Stain

After 10 days of coculturing, osteoclast formation was studied by tartrate-resistant acid phosphatase (TRACP) staining according to the manufacturer's instructions (Enzyme-Labeled Fluorescence, ELF-97 Endogenous phosphatase detection kit, Thermo Fisher) [48]. After fixation, cells were incubated for 15 minutes at 37°C with ELF-97 phosphatase substrate working solution (7.4 mM tartrate, 1.1 mM sodium nitrite, 110 mM acetate solution, and 150 *μ*M ELF-97, all from Thermo Fisher). After one wash with PBS, cells were counterstained with the permeable nuclear dye 4′,6-diamidino-2-phenylindole (DAPI, 300 nM, Thermo Fisher) and washed again. Finally, micrographs were taken with a fluorescence microscope (excitation 360-370, emission 420, Leica DFC320; Leica Microsystems). Cells were considered to be osteoclasts when they were TRACP-positive and contained at least three nuclei.

#### 2.3.10. Statistics

All study population data analyses of part A of this study were performed using the SPSS software (Version 25, IBM, Armonk, NY, USA). The study population data of part A of this study were checked for normality using the D'Agostino-Pearson Omnibus normality test. The age of the study population was found not normally distributed and was therefore tested with Mann-Whitney tests for significant differences between controls and periodontitis patients. For the other normally distributed parameters (BMI, number of teeth), statistical analysis was performed using unpaired *t*-tests. Possible differences were tested between groups for categorical variables using the chi-square tests or Fisher exact tests where appropriate. Patient characteristics were presented as medians (age), means (BMI, number of teeth, and clinical data), or as numbers of subjects (categorical data).

Flow cytometry data of both part A and part B of this study were analyzed using GraphPad Prism software (version 6.07, La Jolla, CA, USA). Flow cytometry data were tested for normality using the D'Agostino-Pearson Omnibus normality tests and found to be normally distributed. Flow cytometry data of part A were compared with unpaired *t*-tests. Flow cytometry data of part B were compared with paired one-way ANOVA between different conditions. In general, flow cytometry data were presented as means ± standard error of the mean (SEM). Differences were considered significant at *p* < 0.05.

## 3. Results

### 3.1. Study Population of Part A

In part A of our study, we investigated RANKL expression by cPMNs and oPMNs from healthy controls and periodontitis patients. Detailed information about the study population is provided in Table 1. This study population consisted of 13 controls and 9 periodontitis patients with a median age of 45 and 49, respectively. The study population consisted of mainly Europeans. More than half of the patient population smoked, while 76.9% did not smoke in the control group. The mean BMI in the patient group was significantly higher than the control group (*p* = 0.008).

### 3.2. RANKL Expression on cPMNs and oPMNs from Healthy Controls and Periodontitis Patients

**Figure 1:**
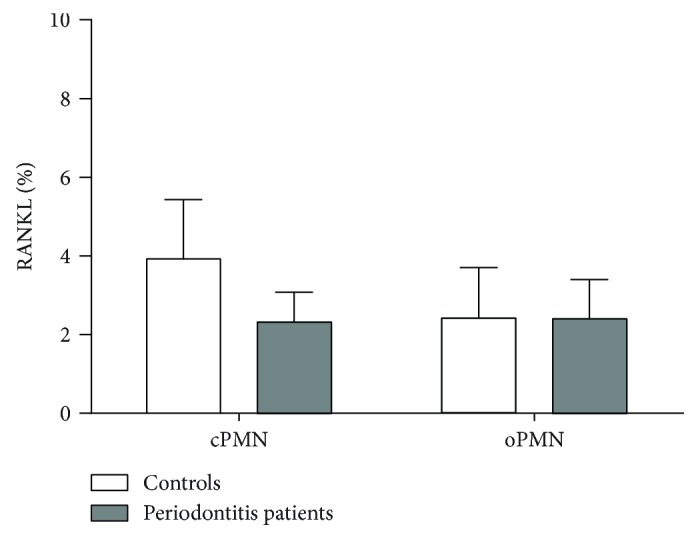
RANKL expression on (unstimulated) PMNs from controls and periodontitis patients. RANKL expression was measured on circulatory (cPMNs) and oral PMNs (oPMNs) from healthy controls (white bars) and periodontitis patients (grey bars). Bars represent the percentage of RANKL expression corrected for IgG isotype control expression on CD66b-positive PMNs. Whiskers demonstrate standard errors of means. The employed gating strategy is shown in Supplementary [Supplementary-material supplementary-material-1]. No significant differences were found between conditions (unpaired *t*-test). *n* = 13 controls, *n* = 9 periodontitis patients.

We measured RANKL expression on cPMNs from healthy controls and found 3.9 ± 1.5% expression (mean ± SEM, Figure 1, white bar). RANKL expression by cPMNs from periodontitis patients (grey bar) was 2.3 ± 0.8%, which did not significantly differ from controls.

Next, we studied the role of oPMNs that have migrated through the periodontium and oral mucosal tissues. It is known that oPMNs have a hyperactive phenotype due to their transmigration through the oral mucosal tissues [35, 36]. We, therefore, hypothesized that oPMNs could express RANKL as they may have been activated by bacteria in the sulcus and oral cavity [49]. We measured RANKL expression on oPMNs from controls and periodontitis patients and found that on average 2.4 ± 1.3% and 2.4 ± 1.0% RANKL was measured on oPMNs from controls and periodontitis patients, respectively (Figure 1). No statistically significant difference in RANKL expression was found on oPMNs between these groups.

### 3.3. LPS Induces Limited RANKL Expression on cPMNs

**Figure 2:**
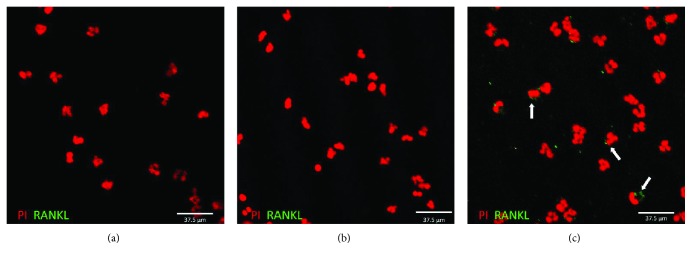
Immunohistochemical visualization of RANKL expression on LPS-stimulated cPMNs. Unstimulated (a) and LPS-stimulated (b, c) cPMNs were stained with the nuclear stain propidium iodide (PI, visualized in red) and RANKL (visualized in green). Unstimulated PMNs do not express RANKL (a). Isotype staining of stimulated cPMNs did not show any green signal (b). RANKL expression is shown here on stimulated cPMNs (indicated with arrows in (c)). Scale bars represent 37.5 *μ*m. Here, representative micrographs of 3 independent experiments are shown. PI is visualized as red fluorescence and RANKL is visualized as green fluorescence.

Based on previous research [37] and to further substantiate the findings from part A of our study, the experiments conducted in part B of this study were designed to further investigate the possible role of cPMNs in the induction of osteoclastogenesis. We investigated whether LPS-stimulated cPMNs expressed RANKL and thus could stimulate osteoclastogenesis. Immunohistochemical staining was performed to visualize RANKL expression on unstimulated and stimulated cPMNs. After stimulation with LPS (100 ng/mL, 24 h), cPMNs were stained for RANKL. Nuclear staining with PI was performed to locate cPMNs. Accordingly, typical PMN lobule nuclei are shown in red (Figure 2). Unstimulated cPMNs did not show any RANKL expression (Figure 2(a), green signal absent), and RANKL expression was absent for the IgG_2b_ isotype control staining of LPS-stimulated cPMNs (Figure 2(b), green signal absent). As shown in the micrographs of Figure 2(c), LPS stimulation induced low levels of RANKL expression of the observed cPMNs (green signal, indicated by arrows, Figure 2(c)). Since RANKL expression was not homogeneously expressed on stimulated cPMNs, we next quantified RANKL expression using flow cytometry.

### 3.4. cPMNs Express Limited Levels of RANKL after LPS Stimulation

**Figure 3:**
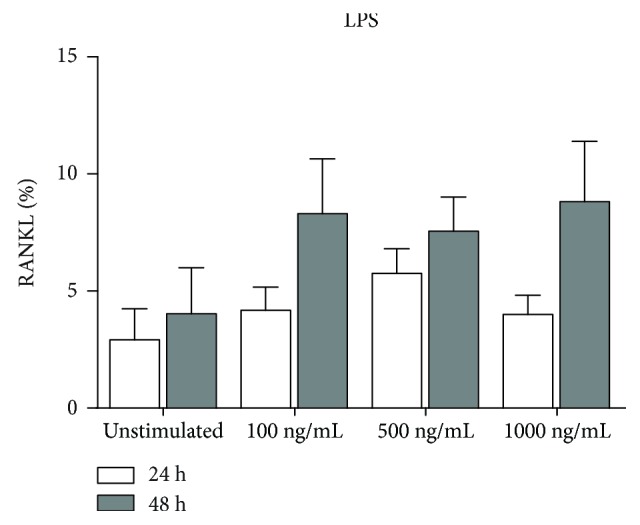
RANKL expression on LPS-stimulated cPMNs. Percentages of RANKL expression by cPMNs after 24 (white bars) and 48 hours (grey bars). Different stimulatory conditions are shown on the *x*-axis. Percentages (mean + standard error of means, *n* = 3) of RANKL expression on live, CD66b-positive cells are shown on the *y*-axis. Overall, no significant differences (paired one-way ANOVA) were observed between unstimulated and stimulated conditions. The employed gating strategy is shown in Supplementary [Supplementary-material supplementary-material-1].

After 24 and 48 hours of stimulation with different concentrations of LPS, the percentages of cPMNs expressing RANKL were measured using flow cytometry. Percentages of RANKL expression by unstimulated and stimulated cPMNs after 24 and 48 hours are shown in Figure 3. After stimulation, no significant increase in the percentage of RANKL expression was found for cPMNs stimulated with 100, 500, or 1,000 ng/mL of LPS (Figure 3). This was the case for both 24- and 48-hour stimulations.

### 3.5. LPS-Stimulated cPMNs Do Not Stimulate Osteoclastogenesis

**Figure 4:**
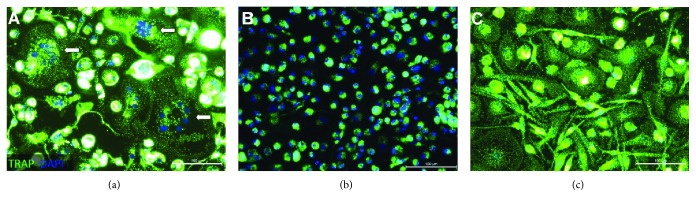
LPS-activated cPMNs do not stimulate osteoclastogenesis in cocultures with osteoclast precursors. Cells were stained for tartrate-resistant acid phosphatase (TRACP, green) and counterstained with the permeable nuclei dye 4′,6-diamidino-2-phenylindole (DAPI, blue). (a) Preosteoclasts (monocytes) were cultured for 10 days with M-CSF and RANKL. Formed osteoclasts are indicated with arrows. (b) Preosteoclasts (monocytes) were cultured for 10 days with M-CSF and unstimulated cPMNs. No osteoclasts were formed in this condition. (c) Preosteoclasts (monocytes) were cocultured with LPS-activated (100 ng/mL, 48 hours) cPMNs. In this condition, no osteoclasts were observed. Representative micrographs of three independent experiments are shown. Scale bars represent 100 *μ*m.

Despite the low levels of RANKL expression after LPS stimulation, it remains possible that these LPS-activated and fixated cPMNs could stimulate osteoclastogenesis. Therefore, we studied osteoclastogenesis directly by coculturing (stimulated) fixated cPMNs with monocytes as preosteoclasts. Control conditions (monocytes cultured with M-CSF and RANKL) showed multinucleated and TRACP-positive cells (indicated with arrows, Figure 4(a)). Next, unstimulated cPMNs were cultured with M-CSF-treated monocytes (Figure 4(b)), showing TRACP-positive mononuclear cells. Neither unstimulated (Figure 4(b)) nor LPS-stimulated cPMNs (Figure 4(c)) lead to the formation of osteoclasts without the addition of RANKL. Noteworthily, the condition containing LPS-stimulated cPMNs (Figure 4(c)) shows elongation of monocytes with relatively big nuclei in comparison to the control condition with unstimulated cPMNs (Figure 4(b)).

### 3.6. Minimal RANKL Expression by cPMNs after Stimulation with IL-6 and TNF-*α*

**Figure 5:**
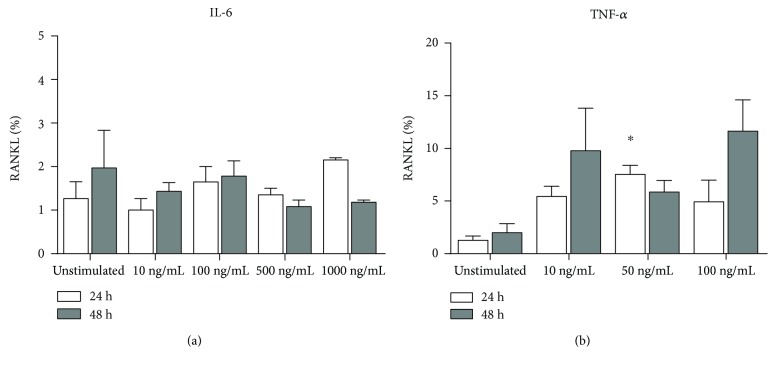
RANKL expression on IL-6- or TNF-*α*-stimulated cPMNs. Percentages of RANKL expression by cPMNs stimulated with IL-6 (a) or TNF-*α* (b) after 24 (white bars) and 48 (grey bars) are shown on the *y*-axes. Concentrations of stimulants are shown on the *x*-axes. Percentages (mean + standard error of means, *n* = 3) of RANKL expression on live, CD66b-positive cells are shown on the *y*-axes. Overall, no significant differences (paired one-way ANOVA) were observed between unstimulated and IL-6-stimulated conditions. The significant difference (^∗^*p* < 0.05) was compared (paired one-way ANOVA) to the unstimulated condition. The employed gating strategy is shown in Supplementary [Supplementary-material supplementary-material-1].

Since LPS induced RANKL expression in cPMNs to a very limited extent, we tested whether proinflammatory cytokines IL-6 and TNF-*α*, known to induce RANKL expression [16, 18], could contribute to an increased RANKL expression of cPMNs [38, 50]. cPMNs were tested for RANKL expression after 24 and 48 hours of stimulation with different concentrations of IL-6 and TNF-*α*. Percentages of RANKL expression by cPMNs after IL-6 stimulation are shown in Figure 5(a). On average, between 0.5 and 2.2% (min–max) cPMNs expressed RANKL after 24 and 48 hours of stimulation with IL-6. This was not significantly different from unstimulated conditions, irrespective of the different concentrations of IL-6 used. Percentages of RANKL expression by cPMNs after TNF-*α* stimulation are shown in Figure 5(b). Stimulation of cPMNs with 50 ng/mL TNF-*α* induced significantly higher (*p* = 0.0364, paired one-way ANOVA) RANKL expression after 24 hours (Figure 5(b)). However, other conditions and concentrations did not differ statistically from unstimulated conditions.

### 3.7. IL-6- and TNF-*α*-Stimulated cPMNs Do Not Stimulate Osteoclastogenesis

**Figure 6:**
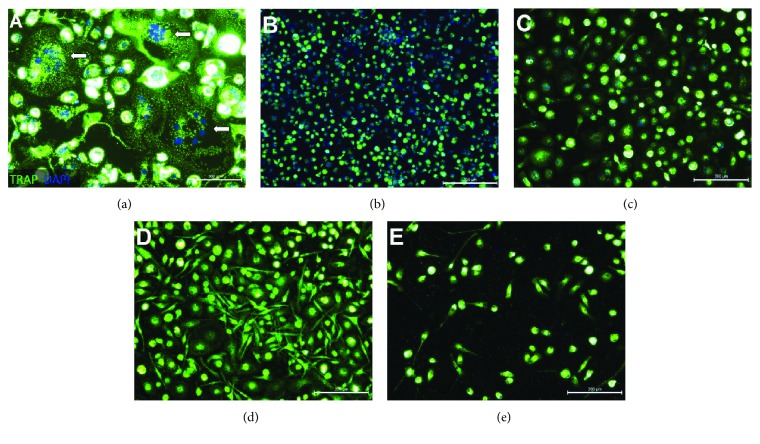
Activated cPMNs do not stimulate osteoclastogenesis in cocultures with osteoclast precursors. Nuclei are stained with DAPI (visualized in blue), and TRACP expression is shown in green. Preosteoclasts (monocytes) were cultured for 10 days with RANKL and M-CSF. (a) TRACP-positive, multinucleated cells were formed (depicted by arrows). (b) Monocytes were cultured with unstimulated cPMNs. (c) Monocytes were cultured with stimulated cPMNs (IL-6, 100 ng/mL for 48 hours) and did not differentiate into TRACP-positive multinucleated cells. (d) Monocytes were cocultured with TNF-*α*-stimulated cPMNs (50 ng/mL for 24 hours) and did not differentiate into osteoclasts. (e) Monocytes were cocultured with TNF-*α*-stimulated cPMNs (50 ng/mL, 48 hours) and did not differentiate into osteoclasts. Representative micrographs of three independent experiments are shown. Scale bars represent 200 *μ*m.

We hypothesized that cytokine-stimulated cPMNs would induce osteoclastogenesis. In order to test our hypothesis, TNF-*α*- or IL-6-stimulated cPMNs were cocultured with monocytes as preosteoclasts in order to investigate osteoclastogenesis. Cocultures of stimulated cPMNs and monocytes after 10 days are shown in Figure 6. Control conditions contained osteoclasts cultured for 10 days with M-CSF and RANKL (Figure 6(a), indicated with arrows). All other conditions were cultured with M-CSF and without RANKL. Hypothetically, osteoclastogenesis stimuli could originate from stimulated activated cPMNs. Thus, monocytes were cocultured with unstimulated cPMNs (Figure 6(b)), IL-6-stimulated cPMNs (100 ng/mL, Figure 6(c)), or TNF-*α*-stimulated cPMNs (50 ng/mL for 24 hours (Figure 6(d)), 50 ng/mL for 48 hours (Figure 6(e))), in addition to M-CSF which was present in all conditions. No osteoclasts were observed in conditions with either stimulated (Figures 6(c)–6(e)) or unstimulated (Figure 6(b)) cPMNs. TNF-*α*-stimulated cPMNs gave rise to more elongated monocytes when cultured together with M-CSF (Figures 6(d) and 6(e)). In conclusion, our results demonstrate that stimulated cPMNs express RANKL in a limited fashion; however, under the current conditions, no osteoclastogenesis was induced.

## 4. Discussion

Osteoclast-mediated resorption of the tooth-adjacent alveolar bone, the hallmark of periodontitis progression, is accompanied by chronic inflammatory cell infiltration of the periodontal soft tissues. PMNs are the predominant innate immune responders that infiltrate inflammatory lesions, and they have been shown to play key roles in chronic inflammatory conditions and in the regulation of immune responses [51]. Part A of this study is aimed at investigating the cellular expression of RANKL by cPMNs and oPMNs taken from both controls and periodontitis patients. Of note, previous studies have shown that periodontal bacteria in intimate contact with the ulcerated epithelium can infiltrate the bloodstream [52, 53]. We, therefore, hypothesized that cPMNs from periodontitis patients express more RANKL than those sourced from controls. However, we found that RANKL expression was not higher in cPMNs from periodontitis patients with chronically inflamed gingival tissues. These data show that cPMNs from periodontitis patients are not per se primed to contribute to osteoclastogenesis via the RANKL pathway.

In cases of chronic inflammation of the periodontium, such as in periodontitis, an increased influx of oPMNs extravasate to the oral cavity [35]. The oral cavity, a microorganism-rich ecosystem, harbors over 700 different species of colonizing bacteria, which possibly prime and activate oPMNs [54]. oPMNs were shown to have a hyperactive phenotype by their hyperactive ROS and NET production [36, 55]. Evidently, oPMNs are more mature cells than cPMNs with exhausted chemotactic capacities due to their transendothelial extravasation, oral transepithelial migration, and exposure to the oral biofilm [36, 40]. Moreover, LPS has been shown to induce RANKL expression [20]. As such, we hypothesized that oPMNs could express more RANKL than cPMNs which originate from the nearly sterile circulatory system [54]. Notwithstanding this, the aforementioned differences did not impact the minimal levels of RANKL expression in both the cPMNs and oPMNs from healthy controls. In periodontitis, a pathogenic imbalance of the oral ecosystem occurs [56]. This, coupled with a persistent immune activation maintained by their inability to eliminate pathogens, causes an aberrant inflammatory response triggering the secretion of important molecular mediators of inflammation, including inflammatory cytokines (such as TNF-*α* and IL-6). These inflammatory cytokines, in turn, can activate oPMNs to express more RANKL. We, therefore, hypothesized that levels of RANKL expression would be higher in oPMNs from periodontitis patients. However, no significant difference was found between the RANKL expression levels of oPMNs and cPMNs originating from either the patient or control groups. Collectively, our results demonstrate that neither contact with bacteria (in oPMN samples) nor chronic gingival inflammation (in periodontitis patients) induces mRANKL expression on cPMNs or oPMNs.

Part B of this study was performed to further investigate and refine the findings of Chakravarti et al. [37]. Chakravarti et al. demonstrated that LPS-stimulated cPMNs have the potential to express RANKL and thereby induce osteoclastogenesis [37]. Despite performing exactly the same protocols (using corresponding chemicals and antibodies), in the same laboratory with the same equipment as described by Chakravarti et al., we were not able to reproduce these findings. They reported that surface RANKL was expressed by less than 5% of unstimulated cPMNs, while on average 23 ± 7% of the LPS-activated cPMNs expressed RANKL. In the current study, we demonstrate that cPMNs expressed lower levels (6.48 ± 0.72%, mean expression ± SEM) of RANKL after 24 or 48 hours of stimulation with LPS. Flow cytometry data is commonly reported as the percentage of cells expressing a certain molecule of interest. In the current case, it is of importance to determine the intensity of this expression by reporting the geometric mean fluorescence intensity (gMFI) of this population given that the intensity of expression can differ between the same numbers of cells in different populations [57]. However, in our studies, the number of events (i.e., PMNs) expressing RANKL, and thus the size of our population of interest, was too small to determine the gMFI. Therefore, the quantification of RANKL expression was solely reported as a percentage of the whole PMN population.

Although RANKL is expressed in three different forms in humans, we solely investigated mRANKL expression by PMNs since it was previously shown that supernatants of cultured activated cPMNs did not contain any sRANKL (detection limit: 15 pg/mL) and no resorption was observed after coculturing preosteoclasts with a conditioned medium of activated PMNs which demonstrates that (stimulated) cPMNs are incapable of producing sRANKL [37]. Nevertheless, it could still be possible that PMNs secrete sRANKL via extracellular vesicles which are membrane-derived vesicles produced in response to various inflammatory stimuli during inflammatory processes [58]. Since it is known that cPMNs do not release sRANKL, effective cell surface interactions require direct cell-cell contact between preosteoclasts and cPMNs. cPMNs have been shown to interact with monocytes [59], and they adhere to osteoclasts after stimulation with LPS [37]. In our study, we did not observe adherence of cPMNs to monocytes; therefore, a direct cell-cell contact, needed for PMN-mRANKL presentation to monocyte-RANK, probably hardly occurred. This, as well as the low levels of RANKL, could be one of the conceivable reasons why no osteoclasts were detected in our coculture experiments.

In our experimental setup, cPMNs were incapable of inducing osteoclastogenesis when fixed and cocultured with preosteoclasts for 10 days. This correlated with a limited RANKL expression, suggesting that the stimuli LPS, TNF-*α*, and IL-6 over the range used were insufficient to activate osteoclastogenesis, either through RANKL or other osteoclastogenesis pathways. Also, it could be that the fixed RANKL was incapable of interacting with the RANK present on osteoclast precursor cells. RANKL dependency was assessed by others by adding OPG to these assays, which was shown to inhibit osteoclast formation [38, 50]. However, the main finding of part B of our study was that cPMNs stimulated with LPS, IL-6, or TNF-*α* did not induce osteoclastogenesis. Since no osteoclasts were formed, the usefulness to perform such experiments with OPG is lacking.

PMNs and T and B cells populate inflamed periodontal lesions [23]. However, the role of PMNs in osteoclastogenesis remains unclear. Riegel and colleagues reported that cPMNs contain preformed RANK, stored in secretory vesicles and specific granules, which can be translocated to the cells' membrane after 24 hours of stimulation with LPS or TNF-*α* [60]. These RANK-positive PMNs, in turn, can be activated via RANKL, in a likely autocrine manner as others have demonstrated that cPMNs express RANKL [37]. However, Riegel et al. investigated the stimulation of cPMNs in whole blood cell cultures. Thus, in the latter study, cPMNs were stimulated in the presence of other immune cells such as monocytes and B and T cells, which could potentially influence the induction of RANK. To support this affirmation, similar results have been shown in other nonpure culture systems such as monocytes in the presence of tooth-associated fibroblasts, in which case the presence of other leukocytes induced the production of (pro-)inflammatory cytokines [5, 22].

PMNs are short-living cells with an estimated half-life of 6-8 hours, which remain in the circulatory system for a few hours before they extravasate into surrounding tissues [61]. Poubelle and colleagues demonstrated that cPMNs incubated for 3 days with medium containing TNF-*α* expressed RANKL and maintained their viability [38]. Despite the use of GM-CSF, a cytokine which improves PMN viability [62], the majority of cPMNs went into apoptosis after 24 to 48 hours of incubation in the present study. To overcome this problem, discontinuous Percoll gradient centrifugation was performed in our experiments to enrich viable cPMNs prior to fixation and culturing with preosteoclasts [47]. Despite the selection of viable cPMNs before fixation, we were not able to find an effect on osteoclast formation.

A challenge in osteoclastogenesis assays is the long duration (minimal 10 days) of these cultures while PMNs are short-lived cells. To overcome this issue, we fixed PMNs to ensure enduring surface expression of RANKL. Performing these experiments with oPMNs was not feasible due to the high numbers of (stimulated) PMNs needed for these experiments (>150 million cPMNs per experiment before stimulation); in our experiments, only ~10% viable cPMNs were obtained after stimulation and discontinuous Percoll gradient centrifugation. Isolating such high numbers oPMNs is not feasible since only approximately 30,000 oPMNs arrive in the oral cavity per minute of a periodontally healthy individual. In contrast to our *in vitro* osteoclastogenesis assays, in *in vivo* situations, PMNs are constantly recruited to the site of inflammation. Therefore, coculturing preosteoclasts with daily fresh additions of (stimulated) cPMNs or oPMNs would be a suggestion for future research to investigate the possible role of PMNs in osteoclastogenesis.

## 5. Conclusion

In conclusion, we report that, in contrast to the study of Chakravarti et al. [37], stimulated cPMNs did not directly stimulate osteoclastogenesis. Furthermore, RANKL expression was not significantly higher on cPMNs and oPMNs originating from periodontitis patients than from controls. Based on our current results, it remains unclear whether PMNs play a role in providing signals to trigger monocytes into the formation of osteoclasts and thus directly activate pathological bone resorption such as present in periodontitis.

## Figures and Tables

**Table 1:** Characteristics of the study population for part A of this study.

	Controls (*n* = 13)	Periodontitis (*n* = 9)	*p* value
Age (years)	45 (26-66)	49 (29-63)	0.5011
Sex (male/female)	6/7	5/4	0.665
Ethnicity (European/non-European)	9/4	6/3	1.000
Smoking (10+ per month/10- per month/not last year)	1/2/10	6/0/3	0.079
Medication (currently using/not currently using)	10/3	2/7	0.027^∗^
Body mass index (kg/m^2^)	23.3 ± 2.1	26.9 ± 3.4	0.008^∗^
Number of teeth	28 ± 3	25 ± 4	0.0405^∗^
#teeth with >50% bone loss	N/A	10.7 ± 6.4	N/A
Sites with plaque (%)	−^a^	64.9 ± 35.9	N/A
Sites with bleeding on probing (%)	−^a^	74.0 ± 14.1	N/A
Probing pocket depth (mm)	−^a^	4.5 ± 2.5	N/A
Pockets ≥ 5 mm (%)	−^a^	53.6 ± 17.5	N/A

Age is presented as medians (range: min–max). Other data are presented as means ± standard deviations or as absolute numbers of subjects. *p* values were calculated with the Mann-Whitney tests (for age), unpaired *t*-tests (for BMI and number of teeth), or chi-square test (Fisher exact tests where appropriate) for categorical data. ^∗^Statistically significant different (*p* < 0.05). ^a^Data were not available for controls since no full mouth periodontal chart has been made. For periodontitis patients, a full mouth periodontal chart was available in the dental records. Abbreviation: N/A = not applicable.

## Data Availability

The flow cytometry data used to support the findings of this study (parts A and B) are available from the corresponding author upon request. The clinical data used to support the findings of part A of this study have not been made available in order to protect patient privacy.

## Abbreviations

BSA: Bovine serum albumin
CD: Cluster of differentiation
DAPI: 4′,6-Diamidino-2-phenylindole
DMEM: Dulbecco's minimal essential medium
*E.coli*: *Escherichia coli*
ELF: Enzyme-labeled fluorescence
GM-CSF: Granulocyte-macrophage colony-stimulating factor
HBSS: Hanks' balanced salt solution
HEPES: 4-(2-Hydroxyethyl)-1-piperazineethanesulfonic acid
IL: Interleukin
LPS: Lipopolysaccharide
M-CSF: Macrophage colony-stimulating factor
MQ: Milli-Q
NF-*κ*B: Nuclear factor kappa B
RANKL: Receptor activator of NF-*κ*B ligand
RT: Room temperature
TNF-*α*: Tumor necrosis alpha
TRACP: Tartrate-resistant acid phosphatase
PBL: Peripheral blood lymphocytes
PBMC: Peripheral blood mononuclear cells.

## Supplementary Materials

Supplementary Materials The gating strategy for flow cytometry experiments is available in the supplementary data.

## References

[1] Hienz S. A., Paliwal S., Ivanovski S. (2015). Mechanisms of bone resorption in periodontitis. *Journal of Immunology Research*.

[2] Slots J. (2017). Periodontitis: facts, fallacies and the future. *Periodontology 2000*.

[3] Asagiri M., Takayanagi H. (2007). The molecular understanding of osteoclast differentiation. *Bone*.

[4] Boyle W. J., Simonet W. S., Lacey D. L. (2003). Osteoclast differentiation and activation. *Nature*.

[5] Bloemen V., Schoenmaker T., de Vries T. J., Everts V. (2010). Direct cell–cell contact between periodontal ligament fibroblasts and osteoclast precursors synergistically increases the expression of genes related to osteoclastogenesis. *Journal of Cellular Physiology*.

[6] Kawai T., Matsuyama T., Hosokawa Y. (2006). B and T lymphocytes are the primary sources of RANKL in the bone resorptive lesion of periodontal disease. *The American Journal of Pathology*.

[7] Sokos D., Everts V., De Vries T. J. (2015). Role of periodontal ligament fibroblasts in osteoclastogenesis: a review. *Journal of Periodontal Research*.

[8] Graves D. T., Alshabab A., Albiero M. L. (2018). Osteocytes play an important role in experimental periodontitis in healthy and diabetic mice through expression of RANKL. *Journal of Clinical Periodontology*.

[9] De Vries T. J., Huesa C. (2019). The osteocyte as a novel key player in understanding periodontitis through its expression of RANKL and sclerostin: a review. *Current Osteoporosis Reports*.

[10] Schramek D., Sigl V., Penninger J. M. (2011). RANKL and RANK in sex hormone-induced breast cancer and breast cancer metastasis. *Trends in Endocrinology and Metabolism*.

[11] Lamont R. J., Koo H., Hajishengallis G. (2018). The oral microbiota: dynamic communities and host interactions. *Nature Reviews. Microbiology*.

[12] Zhang D., Chen L., Li S., Gu Z., Yan J. (2008). Lipopolysaccharide (LPS) of *Porphyromonas gingivalis* induces IL-1*β*, TNF-*α* and IL-6 production by THP-1 cells in a way different from that of *Escherichia coli* LPS. *Innate Immunity*.

[13] Kocgozlu L., Elkaim R., Tenenbaum H., Werner S. (2009). Variable cell responses to P. gingivalis lipopolysaccharide. *Journal of Dental Research*.

[14] Scheres N., Laine M. L., Sipos P. M. (2011). Periodontal ligament and gingival fibroblasts from periodontitis patients are more active in interaction with Porphyromonas gingivalis. *Journal of Periodontal Research*.

[15] Scheres N., De Vries T. J., Brunner J., Crielaard W., Laine M. L., Everts V. (2011). Diverse effects of Porphyromonas gingivalis on human osteoclast formation. *Microbial Pathogenesis*.

[16] Steeve K. T., Marc P., Sandrine T., Dominique H., Yannick F. (2004). IL-6, RANKL, TNF-alpha/IL-1: interrelations in bone resorption pathophysiology. *Cytokine & Growth Factor Reviews*.

[17] Kurihara N., Bertolini D., Suda T., Akiyama Y., Roodman G. D. (1990). IL-6 stimulates osteoclast-like multinucleated cell formation in long term human marrow cultures by inducing IL-1 release. *Journal of Immunology*.

[18] Mori T., Miyamoto T., Yoshida H. (2011). IL-1*β* and TNF*α*-initiated IL-6-STAT3 pathway is critical in mediating inflammatory cytokines and RANKL expression in inflammatory arthritis. *International Immunology*.

[19] Souza P. P. C., Lerner U. H. (2013). The role of cytokines in inflammatory bone loss. *Immunological Investigations*.

[20] Kassem A., Henning P., Lundberg P., Souza P. P. C., Lindholm C., Lerner U. H. (2015). *Porphyromonas gingivalis* stimulates bone resorption by enhancing RANKL (receptor activator of NF-*κ*B ligand) through activation of toll-like receptor 2 in osteoblasts. *Journal of Biological Chemistry*.

[21] Sokos D., Scheres N., Schoenmaker T., Everts V., De Vries T. J. (2014). A challenge with Porphyromonas gingivalis differentially affects the osteoclastogenesis potential of periodontal ligament fibroblasts from periodontitis patients and non-periodontitis donors. *Journal of Clinical Periodontology*.

[22] Moonen C. G. J., Alders S. T., Bontkes H. J. (2018). Survival, retention, and selective proliferation of lymphocytes is mediated by gingival fibroblasts. *Frontiers in Immunology*.

[23] Dutzan N., Konkel J. E., Greenwell-Wild T., Moutsopoulos N. M. (2016). Characterization of the human immune cell network at the gingival barrier. *Mucosal Immunology*.

[24] Teeuw W. J., Gerdes V. E. A., Loos B. G. (2010). Effect of periodontal treatment on glycemic control of diabetic patients: a systematic review and meta-analysis. *Diabetes Care*.

[25] Teeuw W. J., Slot D. E., Susanto H. (2014). Treatment of periodontitis improves the atherosclerotic profile: a systematic review and meta-analysis. *Journal of Clinical Periodontology*.

[26] Chistiakov D. A., Orekhov A. N., Bobryshev Y. V. (2016). Links between atherosclerotic and periodontal disease. *Experimental and Molecular Pathology*.

[27] Lockhart P. B., Bolger A. F., Papapanou P. N. (2012). Periodontal disease and atherosclerotic vascular disease: does the evidence support an independent association?: a scientific statement from the American Heart Association. *Circulation*.

[28] Leliefeld P. H. C., Koenderman L., Pillay J. (2015). How neutrophils shape adaptive immune responses. *Frontiers in Immunology*.

[29] Nicu E. A., Loos B. G. (2016). Polymorphonuclear neutrophils in periodontitis and their possible modulation as a therapeutic approach. *Periodontology 2000*.

[30] Borregaard N., Sørensen O. E., Theilgaard-Mönch K. (2007). Neutrophil granules: a library of innate immunity proteins. *Trends in Immunology*.

[31] Tecchio C., Micheletti A., Cassatella M. A. (2014). Neutrophil-derived cytokines: facts beyond expression. *Frontiers in Immunology*.

[32] Mori G., D'Amelio P., Faccio R., Brunetti G. (2013). The interplay between the bone and the immune system. *Clinical and Developmental Immunology*.

[33] Rijkschroeff P., Loos B. G., Nicu E. A. (2017). Impaired polymorphonuclear neutrophils in the oral cavity of edentulous individuals. *European Journal of Oral Sciences*.

[34] Nicu E. A., Rijkschroeff P., Wartewig E., Nazmi K., Loos B. G. (2018). Characterization of oral polymorphonuclear neutrophils in periodontitis patients: a case-control study. *BMC Oral Health*.

[35] Fine N., Hassanpour S., Borenstein A. (2016). Distinct oral neutrophil subsets define health and periodontal disease states. *Journal of Dental Research*.

[36] Moonen C. G. J., Hirschfeld J., Cheng L., Chapple I. L. C., Loos B. G., Nicu E. A. (2019). Oral neutrophils characterized: chemotactic, phagocytic, and neutrophil extracellular trap (NET) formation properties. *Frontiers in Immunology*.

[37] Chakravarti A., Raquil M. A., Tessier P., Poubelle P. E. (2009). Surface RANKL of Toll-like receptor 4-stimulated human neutrophils activates osteoclastic bone resorption. *Blood*.

[38] Poubelle P. E., Chakravarti A., Fernandes M. J., Doiron K., Marceau A. A. (2007). Differential expression of RANK, RANK-L, and osteoprotegerin by synovial fluid neutrophils from patients with rheumatoid arthritis and by healthy human blood neutrophils. *Arthritis Research & Therapy*.

[39] Zauli G., Corallini F., Bossi F. (2007). Osteoprotegerin increases leukocyte adhesion to endothelial cells both in vitro and in vivo. *Blood*.

[40] Johnstone A. M., Koh A., Goldberg M. B., Glogauer M. (2007). A hyperactive neutrophil phenotype in patients with refractory periodontitis. *Journal of Periodontology*.

[41] Aboodi G. M., Goldberg M. B., Glogauer M. (2011). Refractory periodontitis population characterized by a hyperactive oral neutrophil phenotype. *Journal of Periodontology*.

[42] Ainamo J., Barmes D., Beagrie G., Cutress T., Martin J., Sardo-Infirri J. (1982). Development of the World Health Organization (WHO) community periodontal index of treatment needs (CPITN). *International Dental Journal*.

[43] Tonetti M. S., Claffey N., on behalf of the European Workshop in Periodontology group C (2005). Advances in the progression of periodontitis and proposal of definitions of a periodontitis case and disease progression for use in risk factor research. Group C Consensus report of the 5th European workshop in periodontology. *Journal of Clinical Periodontology*.

[44] Doyle D. J., Garmon E. H. (2018). *American Society of Anesthesiologists Classification (ASA Class)*.

[45] Rijkschroeff P., Jansen I. D. C., Van Der Weijden F. A., Keijser B. J. F., Loos B. G., Nicu E. A. (2016). Oral polymorphonuclear neutrophil characteristics in relation to oral health: a cross-sectional, observational clinical study. *International Journal of Oral Science*.

[46] Lakschevitz F. S., Hassanpour S., Rubin A., Fine N., Sun C., Glogauer M. (2016). Identification of neutrophil surface marker changes in health and inflammation using high-throughput screening flow cytometry. *Experimental Cell Research*.

[47] Ren Y., Stuart L., Lindberg F. P. (2001). Nonphlogistic clearance of late apoptotic neutrophils by macrophages: efficient phagocytosis independent of *β*_2_ integrins. *Journal of Immunology*.

[48] Filgueira L. (2004). Fluorescence-based Staining for Tartrate-resistant Acidic Phosphatase (TRAP) in osteoclasts combined with other fluorescent dyes and protocols. *The Journal of Histochemistry and Cytochemistry*.

[49] Meyle J., Chapple I. (2015). Molecular aspects of the pathogenesis of periodontitis. *Periodontology 2000*.

[50] Chakravarti A., Rusu D., Flamand N., Borgeat P., Poubelle P. E. (2009). Reprogramming of a subpopulation of human blood neutrophils by prolonged exposure to cytokines. *Laboratory Investigation*.

[51] Kaplan M. J., Radic M. (2012). Neutrophil extracellular traps: double-edged swords of innate immunity. *Journal of Immunology*.

[52] Pizzo G., Guiglia R., Russo L. L., Campisi G. (2010). Dentistry and internal medicine: from the focal infection theory to the periodontal medicine concept. *European Journal of Internal Medicine*.

[53] Parahitiyawa N. B., Jin L. J., Leung W. K., Yam W. C., Samaranayake L. P. (2009). Microbiology of odontogenic bacteremia: beyond endocarditis. *Clinical Microbiology Reviews*.

[54] Kilian M., Chapple I. L. C., Hannig M. (2016). The oral microbiome – an update for oral healthcare professionals. *British Dental Journal*.

[55] Rijkschroeff P., Gunput S. T. G., Ligtenberg A. J. M., Veerman E. C. I., Loos B. G., Nicu E. A. (2018). Polymorphonuclear neutrophil integrity and functionality are preserved when exposed to saliva. *Archives of Oral Biology*.

[56] Gao L., Xu T., Huang G., Jiang S., Gu Y., Chen F. (2018). Oral microbiomes: more and more importance in oral cavity and whole body. *Protein & Cell*.

[57] Cossarizza A., Chang H. D., Radbruch A. (2017). Guidelines for the use of flow cytometry and cell sorting in immunological studies. *European Journal of Immunology*.

[58] Lorincz A. M., Schutte M., Timar C. I. (2015). Functionally and morphologically distinct populations of extracellular vesicles produced by human neutrophilic granulocytes. *Journal of Leukocyte Biology*.

[59] Prame Kumar K., Nicholls A. J., Wong C. H. Y. (2018). Partners in crime: neutrophils and monocytes/macrophages in inflammation and disease. *Cell and Tissue Research*.

[60] Riegel A., Maurer T., Prior B. (2012). Human polymorphonuclear neutrophils express RANK and are activated by its ligand, RANKL. *European Journal of Immunology*.

[61] Summers C., Rankin S. M., Condliffe A. M., Singh N., Peters A. M., Chilvers E. R. (2010). Neutrophil kinetics in health and disease. *Trends in Immunology*.

[62] Brach M., deVos S., Gruss H., Herrmann F. (1992). Prolongation of survival of human polymorphonuclear neutrophils by granulocyte-macrophage colony-stimulating factor is caused by inhibition of programmed cell death. *Blood*.

