# Supplementary material for: The Possible Role of Neutrophils in the Induction of Osteoclastogenesis
Source: J Immunol Res. 2019 Sep 15;2019:8672604. doi: 10.1155/2019/8672604 (PMC6766092; doi:10.1155/2019/8672604)
Supplement: Supplementary Materials — The gating strategy for flow cytometry experiments is available in the supplementary data. [file 8672604.f1.pdf]

Supplementary data

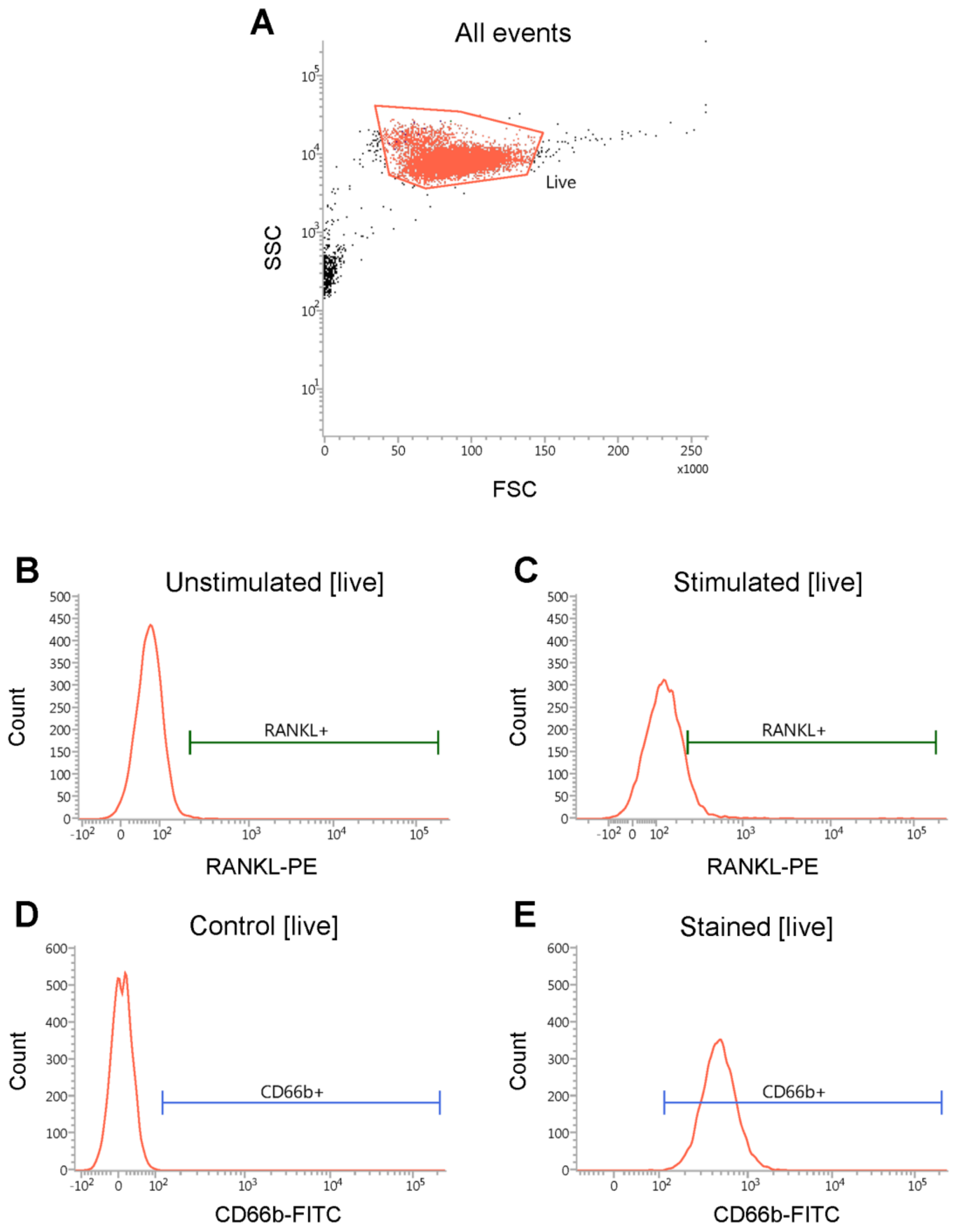

Supplementary Figure 1: Gating strategy for flow cytometry experiments. The antibody combination for flow cytometric experiments was anti-RANKL-PE/CD66b-FITC. Acquisition and analysis were performed on a BD FACSverse flow cytometer using associated FACSuite software. The gating tree was set as follows: **(A)** forward scatter/sideward scatter representing the distribution of cells in the light scatter based on size and intracellular composition, respectively, where the live population is encircled in red. In the [live] population, **(B,C)** RANKL, **(D,E)** CD66b+ cells were identified. **(A)** The RANKL+ gating (green) was set in unstimulated cPMN samples**. (D)** The CD66b+ gating (blue) was set in unstained cPMN samples. Here, representative plots of unstimulated **(B)** and stimulated **(C, D, E)** cPMNs are shown.
